# A study on the impact of catastrophic health expenditure on social stability

**DOI:** 10.3389/fpubh.2026.1799158

**Published:** 2026-04-14

**Authors:** Guowu Huang, Yunzhen Lv

**Affiliations:** Sichuan University, Chengdu, China

**Keywords:** catastrophic health expenditure, health insurance, social fairness perceptions, social identity perceptions, social stability

## Abstract

**Objectives:**

To reveal the impact of catastrophic health expenditure (CHE) on social stability by examining individuals’ sense of social equity and sense of social identity.

**Methods:**

Data from the 2023 Chinese Social Survey and an ordered logit model are used to analyze the impact of CHE on social stability from the dual perspectives of individuals’ sense of social equity and sense of social identity. The individual sense of social equity is constructed using three evaluation dimensions: public healthcare, social security, and urban–rural rights and benefits. Meanwhile, the individual sense of social identity is developed based on three indicators: subjective well-being, life satisfaction, and social trust.

**Results:**

Compared to individuals without CHE, those with CHE report a significantly lower sense of social equity and social identity; their perceptions of public healthcare equity, social security equity, urban–rural rights and benefits equity, as well as subjective well-being, life satisfaction, and social trust are lower by 0.107, 0.132, 0.214, 0.205, 0.340 and 0.165, respectively. Moreover, with the increase in the intensity of catastrophic health expenditure, individuals’ perceptions of public healthcare equity, social security equity, urban–rural rights and benefits equity, along with their subjective well-being, life satisfaction, and social trust decrease by 0.323, 0.292, 0.559, 0.630, 0.980, and 0.608, respectively.

**Conclusion:**

CHE diminishes individuals’ perceptions of social equity and social identity, and a rise in its intensity exerts a further significant impact on social stability. Efforts should focus on optimizing the allocation of medical resources and adjusting fiscal input to advance equity in access to high-quality medical services, balancing gaps in medical insurance benefits and reducing individuals’ medical expense burden, adjusting existing reimbursement policies, exploring the establishment of an income-related out-of-pocket payment ceiling mechanism to control out-of-pocket medical expenses, and avert the occurrence of CHE.

## Introduction

Catastrophic health expenditure (CHE) imposes a heavy medical cost burden on families (1), crowds out residents’ consumption or necessary expenditures, affects family finances, plunges families into economic difficulties (2), and may even lead to poverty because of illness. In 2015, the State Council issued Opinions on the Comprehensive Implementation of Critical Illness Insurance for Urban and Rural Residents, which proposed reducing residents’ out-of-pocket medical expenses and sharing disease risks through a multilevel medical security system to effectively prevent family CHE. According to the World Health Organization’s statistics, the incidence of CHE in China reached 6.94% in 2018 (3), measured as out-of-pocket medical expenses exceeding 25% of household total expenditure or income. Empirical studies using microdata have also shown that although China’s CHE incidence declined from 16.27% in 2012 to 13.88% in 2020 (defined as out-of-pocket medical expenses accounting for 40% of household capacity to pay), the depth and duration of CHE have been on the rise (4), indicating that CHE still poses considerable risks to individuals, households, and social stability.

According to the Sustainable Development Goals (SDGs), CHE occurs when a household’s out-of-pocket medical expenses comprise more than 10% or 25% of total household consumption expenditure or household income (5). This definition involves three core components: household out-of-pocket medical expenses, household capacity to pay, and the threshold ratio between them. Household out-of-pocket medical expenses are medical costs that patients and their families must bear independently after excluding insurance reimbursements. Regarding household capacity to pay, some scholars directly adopt household income or per capita household income (6–8) as the measurement standard, whereas others use total household expenditure or household non-food expenditure (9, 10). Regarding the threshold of the ratio of out-of-pocket medical expenses to household capacity to pay, the most commonly used standards in academic research are 10% (11, 12) of total household expenditure or 40% (13, 14) of household non-food expenditure. In this study, the proportion of household out-of-pocket medical expenses accounting for 10% of total household expenditure is selected as the criterion for determining the occurrence of CHE.

Social stability encompasses multiple dimensions of social life and communal life (15), including social trust, fairness and justice, equal rights, the level of medical services and social security (16), and other aspects. It reflects the living structure of individuals in society and their appropriate state of social participation, and has a strong correlation with individual psychological conditions (17). CHE pushes households into poverty and reduces household consumption below the minimum required level by depleting household assets through income loss and the sale of productive assets (18–20). It directly impacts individual quality of life and well-being (21), lowers life satisfaction and subjective well-being (22), impairs individual mental health (59), and exerts adverse effects on social stability (23). The shock of serious illness intensifies the contradiction between high medical costs and insufficient medical security (24), leading to an overburden of out-of-pocket medical expenses for households, which in turn amplifies individuals’ sense of insecurity and dissatisfaction, thereby undermining social stability (23). Although health insurance can alleviate CHE and the poverty issues arising therefrom to a certain extent (25), disparities in insurance benefits associated with factors such as personal identity, insurance enrollment type, income level, and local fiscal capacity (26–28) have exacerbated inequalities in the incidence of CHE (29, 30). This results in relatively vulnerable groups facing a higher probability of incurring CHE and falling into poverty (31–34), which strengthens individuals’ perceptions of unfairness (35, 36) and is detrimental to social stability and development.

In summary, existing research on CHE and social stability is mostly confined to the separate dimensions of CHE or social stability itself, and fails to integrate CHE with social stability to further explore the impact of CHE on social stability. Even though some scholars have proposed that social stability should be promoted by improving health insurance (37), they have not established a connection among CHE, health insurance, and social stability. Therefore, exploring the impact of CHE on individuals’ social cognition and its ultimate upward effect on social stability, while analyzing the potential causes of such an impact, is of great significance for rethinking and adjusting the existing policy system and advancing social stability.

## Theoretical analysis and research hypotheses

Individuals’ sense of social fairness, as well as social identity and integration, exert a significant impact on social stability (17, 38). Therefore, to explore the impact of CHE on social stability in a more comprehensive manner, both individuals’ sense of social fairness and social identity are adopted to measure the impact of CHE on social stability.

### Catastrophic health expenditure and social fairness perceptions

Social equity perception refers to the perceptions and evaluations of an individual or group regarding whether social resources, access to opportunities, and other related aspects are fairly and reasonably distributed. People’s perceptions of social equity are affected by two factors: objective social equity (i.e., whether the procedures and outcomes of social distribution in terms of resources, opportunities, and other aspects are fair) and individual perceptions. People’s perceptions of social equity are influenced by their own resource endowments, life experiences, and social comparisons, and individual differences lead to variations in how different individuals may perceive the fairness of the same state of social resource distribution and rights protection. From the perspective of resource access, the uneven distribution of high-quality medical resources leads to inequalities in the accessibility and acquisition costs of medical services among individuals (39), which induces disparities in healthcare-seeking behaviors (40). Disadvantaged individuals have to obtain high-quality medical services through means such as cross-regional medical treatment, which increases their healthcare costs and impairs their perceptions of fairness in public medical care. From the perspective of objective social institutional arrangements, due to the low level of institutional overall planning and fragmentation, China’s existing medical insurance system suffers from the dualization of benefits between employee and resident medical insurance, large benefit gaps within resident medical insurance, and the regressive distribution of medical security resources (41). CHE is an important manifestation of medical inequality (42). Under China’s existing institutional arrangements, there exists income-related inequality in the risk of CHE (2, 43). Institutional design leads to disparities in the medical insurance entitlements obtained by different individuals, which in turn results in variations in their risk of incurring CHE and impairs their perceptions of fairness in social security. Moreover, disparities in resource distribution and benefit protection are inextricably linked to the urban–rural dual system; thus, evaluations of public medical care and social security benefits will further extend to perceptions of unfairness in rights and benefits between urban and rural areas.

From the perspective of subjective individual cognition, individual perceptions of social equity depend not only on the objective level of social equity but also on group comparisons. People typically compare their outcomes with those of others. Thus, when individuals perceive their own outcomes to be worse than those of others, they experience relative deprivation (44, 45), consider this gap unreasonable, and develop a sense of unfairness. Compared with groups with higher income and social status, low-income vulnerable groups have a weaker risk-bearing capacity. Owing to their lower household payment capacity, the same medical expenses impose a heavier burden on low-income groups. As medical costs accumulate, low-income households are more likely to cross the threshold and incur CHE, resulting in a higher CHE incidence rate (46). Consequently, households and individuals who experience CHE tend to develop a sense of unfairness, and within a certain range, this sense of unfairness intensifies as the proportion of their out-of-pocket medical expenses rises alongside the increase in their CHE ratio. Based on this, the following hypotheses are proposed:

H1: CHE reduces individuals’ social fairness perceptions.

H2: Individuals’ social fairness perceptions decline as the intensity of catastrophic health expenditure increases.

### Catastrophic health expenditure and social identity perceptions

Individual social identity perception is a crucial factor influencing social cohesion and social stability (47). Individual social identification with society refers to a subjective psychological bonding state between individuals and the groups and society to which they belong. The degree to which individuals’ needs are met greatly shapes their sense of identity with the relevant groups and society; when individuals’ needs are satisfied, they experience higher life satisfaction and well-being, along with a stronger subjective sense of identity with their groups (48). The essence of CHE lies in the fact that out-of-pocket medical expenses rise to exceed a household’s economic affordability, which crowds out the household’s other consumption and impacts its normal consumption and daily life.

In other words, the occurrence of CHE reveals, to a certain extent, that an individual’s demand for medical security in this social context has not been adequately met. Under the shock of CHE, individuals have to bear disease risk, poverty risk, and a heavy economic burden simultaneously. Individuals and their families face considerable uncertainty and vulnerability. To meet the needs of medical treatment, individuals and their households must cut necessary expenses or even resort to harmful measures that undermine family stability, such as borrowing (49). Under such circumstances, compared with the situation before CHE occurred, the living standards of individuals and families decline significantly, making them more likely to develop a strong sense of relative deprivation, which directly affects their well-being, life satisfaction, and level of social trust. Furthermore, as their out-of-pocket medical expense burden increases, these effects intensify. Accordingly, the following hypotheses are proposed:

H3: CHE reduces individuals’ social identity perceptions.

H4: Individuals’ social identity perceptions decrease as the intensity of catastrophic health expenditure increases.

**Figure 1:**
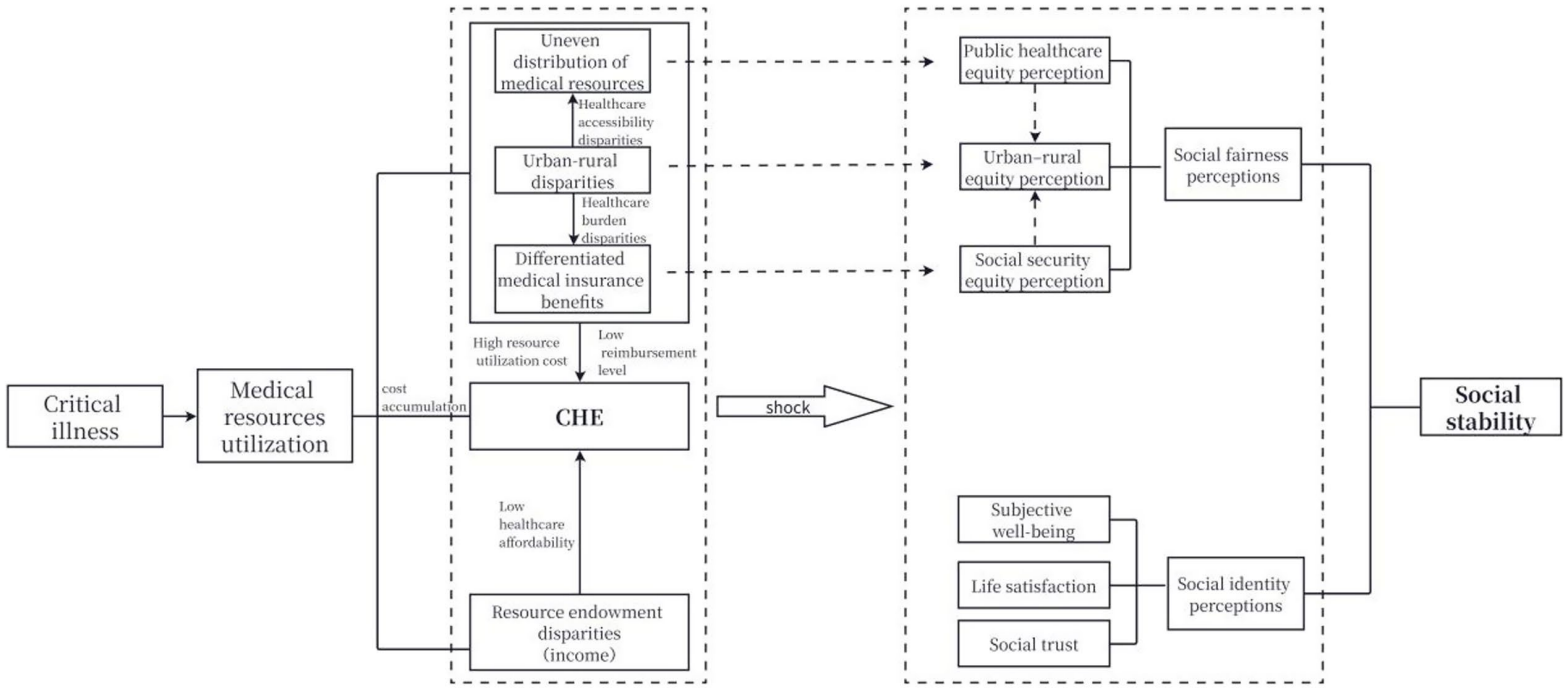
Analytical framework.

Overall, individuals with illnesses have substantial medical needs. To meet their treatment demands, they and their families often choose to seek medical care at higher-level medical institutions, where medical expenses continue to accumulate during the treatment process. Given the same health needs, both the unequal distribution of medical resources and the unfairness of medical insurance benefits, which are linked to urban–rural disparities, together with differences in individual resource endowments such as income, make disadvantaged individuals more likely to cross the threshold of out-of-pocket medical expenses and incur CHE, resulting in considerable economic and psychological pressure. Such disparities in CHE risk lead to perceptions of unfairness toward public medical care, social security, and urban–rural rights. Meanwhile, CHE directly lowers individuals’ quality of life, impairs their well-being, life satisfaction, and social trust, and ultimately exerts an adverse impact on social stability (Figure 1).

## Methods

### Data source

The data used in this study were obtained from the 2023 Chinese Social Survey (CSS) database. Launched by the Institute of Sociology, Chinese Social Sciences in 2005, the CSS is a large-scale national continuous sampling survey project. It covers 31 provinces/autonomous regions/municipalities directly under the Central Government across China, including 156 districts, counties, and cities. The CSS collects data on the public’s labor and employment, family and social life, social attitudes, and other aspects, making it nationally representative.

The CSS 2023 database contains a total of 13,035 observations, to verify the impact of CHE on residents’ perceptions of social equity, this study excluded samples in which key variables, including total household living expenditure, medical expenditure, total household income, household size, evaluation of the fairness of public healthcare, evaluation of the fairness of social security benefits, evaluation of the fairness of rights and benefits between urban and rural areas, and evaluation of the overall social fairness, were recorded as “do not know,” “hard to say,” “unclear,” or “refuse to answer,” or had missing values. Additionally, 1% winsorization was applied to the data to avoid the impact of extreme values on the results. Finally, 8,599 valid samples were retained.

### Variable selection and definitions

#### Dependent variable

To explore the impact of CHE on social stability, this study takes individuals’ social fairness perceptions and social identity perceptions as the dependent variables, respectively, to conduct regression analyses. This study focuses on exploring the impact of CHE on individual perceptions of social equity, which is primarily related to the medical field and arises from residents’ use of public medical services and medical insurance; therefore, the occurrence of CHE will directly affect residents’ perceptions and evaluations of the equity of public medical services and the social security system, including medical insurance. In addition, based on the above analysis, urban–rural gaps exist in the allocation of medical resources and medical security benefits in China. Therefore, an individual’s evaluation of the equity of public medical services and social security caused by CHE will be further transmitted to form an evaluation of the equity of benefits and rights between urban and rural areas. Thus, individual evaluations of the equity of public medical services, social security benefits, and rights and benefits in urban and rural areas were used as explained variables to jointly construct a comprehensive evaluation system for individual perceptions of social equity, which, respectively, correspond to three items on the questionnaire: “What do you think about the equity of public medical services in current social life”; “What do you think about the equity of social security benefits, such as public older adult care, in current social life”; and “What do you think about the equity of rights and benefits between urban and rural areas in current social life?”

For individuals’ social identity perceptions, when CHE occurs, individuals’ perceived satisfaction with their current living conditions diminishes, and they also develop a sense of relative deprivation, which impairs their level of social trust. Thus, this study measures individuals’ social identity perceptions by adopting their evaluations of their own subjective well-being, life satisfaction, and social trust, which correspond, respectively, to the three questions in the questionnaire: “Do you agree with the following statement: Generally speaking, I am a happy person”; “Your satisfaction with life”; and “The level of trust between people.”

#### Explanatory variables

CHE is calculated as the proportion of out-of-pocket medical expenditure relative to a household’s capacity to pay. The medical expenditure data are derived from the following questionnaire item: “Medical and health care expenditure (e.g., expenses for medical treatment, hospitalization, medicine purchase, etc., after deducting the reimbursed portion).” Based on a review of existing literature, this study adopts the threshold that OOP medical expenditure accounts for 10% or more of total household expenditure as the criterion for identifying CHE (11, 50). Specifically, CHE is coded as 1 if the share of OOP medical expenses in total household expenditure is 10% or above, and 0 otherwise. In addition, the ratio of out-of-pocket medical expenditure to total household expenditure is used to measure CHE intensity. For individuals with a ratio below 10%, CHE intensity is set to 0; for others, CHE intensity is equal to their actual ratio of out-of-pocket medical expenditure to total household expenditure.

#### Other variables

CHE reduces the quality of life of individuals and their households, exposes them to high levels of uncertainty and risk, and undermines social stability. Therefore, income level, accessibility to medical services, medical service prices, and type of insurance enrollment are further introduced as mechanism variables to analyze the causes of CHE. Among them, income level is divided into five income groups based on total household income. Accessibility to medical services is measured using the survey item “Did you encounter the problem that the clinic/hospital was too far away when seeking medical care in the past year?.” Medical service prices are measured using the item “Did you encounter the problem that medical expenses were too expensive when seeking medical care in the past year?.” The type of insurance enrollment is determined according to the medical insurance scheme in which the individual is enrolled.

#### Control variables

Referring to existing studies, other factors that influence individual perceptions and evaluations of social equity are selected as control variables (51). Comprehensively considering individual- and household-level attributes and characteristics, the control variables include gender, age, educational level, marital status, ethnicity, and household registration type. Meanwhile, since the proportion of out-of-pocket medical expenses to total household expenditure is adopted as the criterion for identifying CHE in the baseline regression, total household income is also included as a control variable in the baseline regression (Table 1).

**Table 1:** Variable descriptions.

Variable names	Definitions
Public healthcare equity perception (public_healthcare_fairness)	An individual’s evaluation of the degree of fairness in public medical care is coded as follows: “very unfair” = 1, “somewhat unfair” = 2, “somewhat fair” = 3, and “very fair” = 4.
Social security equity perception (social_security_fairness)	An individual’s evaluation of the degree of fairness in social security is coded as follows: “very unfair” = 1, “somewhat unfair” = 2, “somewhat fair” = 3, and “very fair” = 4.
Urban–rural equity perception (urban_rural_fairness)	An individual’s evaluation of the degree of fairness in the rights and security between urban and rural areas is coded as follows: “very unfair” = 1, “somewhat unfair” = 2, “somewhat fair” = 3, and “very fair” = 4.
Subjective well-being (happiness)	An individual’s evaluation of their own subjective well-being, coded as 1, 2, 3, and 4 for extremely unhappy, not very happy, quite happy, and extremely happy, respectively.
Life satisfaction (life_satisfaction)	An individual’s evaluation of the level of life satisfaction, with the satisfaction scale from 1 to 10 evenly divided into five categories: “extremely dissatisfied” = 1, “somewhat dissatisfied” = 2, “neutral” = 3, “somewhat satisfied” = 4, and “extremely satisfied” = 5.
Social trust (social_trust)	An individual’s evaluation of the level of social trust, with the trust scale from 1 to 10 evenly divided into five categories: “extremely distrustful” = 1, “somewhat distrustful” = 2, “neutral” = 3, “somewhat trusting” = 4, and “extremely trusting” = 5.
Catastrophic health expenditure (che)	If the out-of-pocket medical expenses of an individual or household account for 10% or more of the total household expenditure (i.e., out-of-pocket medical expenses / total household expenditure ≥ 10%), it is regarded as the occurrence of catastrophic health expenditure and coded as 1; otherwise, it is coded as 0.
Catastrophic health expenditure intensity (che_int)	This variable is coded as 0 if the share of out-of-pocket medical payments in total household expenditure is less than 10%. If the share is 10% or higher, the actual proportion of out-of-pocket payments is used to represent the intensity of catastrophic health expenditure experienced by the individual and their household.
Catastrophic health expenditure rate (che_rate)	The proportion of individuals who incurred catastrophic health expenditure among all valid samples within the same prefecture- or county-level administrative unit.
Income level (income_level)	Samples are divided into five groups according to total household income: low-income group, lower-income group, middle-income group, higher-income group and high-income group, coded as 1, 2, 3, 4 and 5, respectively.
Access to medical services (accessibility)	Coded as 1, 2, 3 and 4, respectively, for the severity of the problem of clinics/hospitals being too far away: extremely severe, somewhat severe, not very severe, and no such problem.
Medical service prices (price)	Coded as 1, 2, 3 and 4, respectively, for the severity of the problem of excessive medical expenses: extremely severe, somewhat severe, not very severe, and no such problem.
Medical insurance enrollment type (type)	Samples are divided into six groups according to the type of medical insurance enrolled in: Basic Urban Employee Medical Insurance, Basic Urban Resident Medical Insurance, Government Employee Medical Insurance, New Rural Cooperative Medical Insurance (NCMS), Major Disease Insurance, and other types. A value of 1 is assigned if a sample belongs to the corresponding group, and 0 otherwise.
Gender (gender)	Male is coded as 1 and female is coded as 0.
Age (age)	Age is calculated using the following formula: Age = 2025 - Year of Birth.
Educational level (edu)	Educational attainment is coded based on the highest level of education completed, coded as follows: “never attended school” = 1, “primary school” = 2, “junior high school” = 3, “senior high school” = 4, “secondary technical school” = 5, “vocational high school/technical school” = 6, “junior college” = 7, “undergraduate” = 8, “postgraduate (master’s and doctoral)” = 9, and“others” = 10
Marital status (marriage)	Marital status is coded according to an individual’s current marital state, as follows: “unmarried” = 1, “first marriage with spouse” = 2, “remarriage with spouse” = 3, “divorced” = 4, “widowed” = 5, and “cohabiting” = 6.
Ethnicity (ethnic)	Ethnicity is coded as follows: “Han ethnicity” = 1 and “ethnic minorities” = 0.
Household registration type (hukou)	Household registration type is coded based on category as follows: “agricultural household registration” = 1, “non-agricultural household registration” = 2, “residential household registration” = 3, and“others” = 4.
Annual total household income (total_income)	The total income of all household members in the past year.

### Regression model and methods

This study primarily examines the relationship between CHE and social stability, and comprehensively adopts individuals’ social fairness perceptions and social identity perceptions to measure social stability. Since individuals’ social fairness perceptions and social identity perceptions involve multiple dimensions, this study selects individuals’ evaluations of the fairness of public medical services, social security benefits, and rights and benefits between urban and rural areas, as well as their well-being, life satisfaction, and social trust—all of which are closely related to CHE—to comprehensively construct the measurements of individuals’ social fairness perceptions and social identity perceptions. Given that the multiple dependent variables involved are all ordinal variables, and the independent variables, namely CHE and CHE intensity, are a dichotomous variable and a continuous variable, respectively, with reference to previous studies, this study formulates the analytical model using the ordered logit model as follows Equations (1, 2):


$$Y_{i}^{*}=\alpha X_{i}+\varepsilon_{i}\;\;(i=1,2,3\cdots n)$$ (1)

$$Y_{i}=\{\begin{array}{l} \begin{array}{cc} 1 & Y_{i}^{*}\leq k_{1}\\ 2 & k_{1}<Y_{i}^{*}\leq k_{2} \end{array}\\ \begin{array}{cc} \vdots & \\ n & Y_{i}^{*}\leq k_{n-1} \end{array} \end{array}$$ (2)

Where Y_i_* is a latent variable with unobservable specific values, namely individual perceptions of social equity; X_i_ is the explanatory variable, including whether CHE occurs (1 if CHE occurs, 0 otherwise) and the intensity of CHE (0 if no CHE occurs, otherwise the specific proportion of CHE); *α* is the parameter to be estimated. If α is significantly less than 0, it indicates that individuals with CHE have significantly lower perceptions of equity than those without CHE, i.e., CHE reduces residents’ evaluations of Y_i_; ε_i_ is a random error term following the logistic distribution; Y_i_ is an observable variable that reflects individuals’ perceptions of social equity through their evaluation of a specific indicator. This study mainly involves evaluations of the fairness of public medical care, social security, rights, and benefits between urban and rural areas, subjective well-being, life satisfaction, and social trust. All these are observable variables represented by integer values from 1 to 4 or 1 to 5 based on questionnaire data, and k_n_ is the cutoff point of the interval.

In this study, three dependent variables—perceptions of public medical equity, perceptions of social security equity, and perceptions of urban–rural equity—are separately regressed on CHE and CHE intensity. The effect of CHE and CHE intensity on individuals’ perceptions of social equity is measured by synthesizing the regression results of these three independent models. Meanwhile, another three dependent variables—subjective well-being, life satisfaction, and social trust—are separately regressed on CHE and CHE intensity. The effect of CHE and CHE intensity on individual social identity perceptions are measured by synthesizing the regression results of these three independent models. Finally, the impact of CHE on social stability is evaluated by combining its effects on individual social fairness perceptions and social identity perceptions.

To ensure that the regression results can be generalized from the sample to the overall population, sampling probability weights provided by the CSS2023 survey database are applied in all regressions of this study to correct estimation bias caused by the stratified sampling design.

## Results

### Empirical results

#### Descriptive statistics

The analytical sample consists of a total of 8,599 observations, with females accounting for 53.7% and males 46.3%. The overall average age of all samples is 48.05 years, with the minimum age being 22 years and the maximum 77 years. The average total household income stands at 113,320.7 yuan, the average annual total household expenditure at 87,032 yuan, and the average household out-of-pocket medical expenditure at 6,782.659 yuan. In total, 31.8% of the samples incurred CHE. The county/district with the highest CHE incidence rate reaches 68.8%, while the region with the lowest is merely 8.1%, indicating a significant issue of inequality in CHE risk. The descriptive statistical results of the core variables in the sample are presented in Table 2.

**Table 2:** Descriptive statistical results of core variables in the sample.

Variables	Observations	Mean	Standard deviation	Min	Max
Public healthcare equity perception	8,599	2.895	0.775	1	4
Social security equity perception	8,599	2.738	0.871	1	4
Urban–rural equity perception	8,599	2.476	0.901	1	4
Subjective well-being	8,599	3.207	0.724	1	4
Life satisfaction	8,599	4.156	1.032	1	5
Social trust	8,599	3.939	1.026	1	5
Catastrophic health expenditure	8,599	0.318	0.466	0	1
Catastrophic health expenditure intensity	8,599	0.088	0.168	0	0.992
Catastrophic health expenditure rate	8,599	0.318	0.113	0.081	0.688
Income level	8,599	3.048	1.436	1	5
Healthcare accessibility	6,989	3.313	0.851	1	4
Healthcare service prices	6,989	2.617	1.064	1	4
Urban employees’ basic medical insurance	8,599	0.270	0.444	0	1
Urban residents’ basic medical insurance	8,599	0.148	0.355	0	1
Public medical services	8,599	0.214	0.145	0	1
New rural cooperative medical scheme	8,599	0.452	0.498	0	1
Critical illness insurance	8,599	0.035	0.183	0	1
Other medical insurance	8,599	0.021	0.142	0	1
Gender	8,599	1.537	0.499	1	2
Age	8,599	48.053	13.987	20	77
Educational level	8,599	4.271	2.534	1	10
Marital status	8,599	2.072	0.821	1	6
Ethnicity	8,599	0.907	0.291	0	1
Household registration type	8,599	1.582	0.806	1	4
Annual total household income	8,599	113320.7	118927.8	0	672,000
Annual total household expenditure	8,599	87,032	140448.8	111	4,000,000
Health expenditure	8,599	6782.659	19276.85	0	800,000

#### The impact of CHE occurrence on social stability

An ordered logit model is employed to analyze the impact of CHE on individuals’ evaluations of fairness in public medical care, social security, and urban–rural benefits, as well as on their subjective well-being, life satisfaction, and social trust. To ensure the robustness of the results, control variables at the individual and household levels are included in the regression analyses, with the results presented in Tables 3, 4.

**Table 3:** The impact of CHE on individuals’ social fairness perceptions.

Variables	(1)		(2)		(3)	
	Public healthcare equity perception		Social security equity perception		Urban–rural equity perception	
Catastrophic health expenditure	−0.151*** (0.048)	−0.107** (0.049)	−0.184*** (0.047)	−0.132*** (0.048)	−0.289*** (0.045)	−0.214*** (0.046)
Gender		0.126*** (0.044)		−0.016 (0.042)		0.031 (0.042)
Educational level		−0.023* (0.012)		−0.035*** (0.012)		−0.010 (0.012)
Age		−0.015*** (0.002)		−0.019*** (0.002)		−0.017*** (0.002)
Marital status		−0.127*** (0.032)		−0.029 (0.030)		−0.012 (0.029)
Ethnicity		−0.903*** (0.087)		−0.963*** (0.084)		−0.807*** (0.092)
Household registration type		−0.058** (0.028)		−0.022 (0.027)		0.079*** (0.026)
Total income		−0.000 (0.000)		−0.000 (0.000)		0.000 (0.000)
/cut1	−2.945*** (0.050)	−4.741*** (0.175)	−2.250*** (0.038)	−4.305*** (0.168)	−1.797*** (0.034)	−3.180*** (0.171)
/cut2	−1.224*** (0.030)	−2.999*** (0.169)	−0.852*** (0.028)	−2.882*** (0.164)	−0.232*** (0.026)	−1.587*** (0.168)
/cut3	1.380*** (0.031)	−0.321* (0.164)	1.547*** (0.032)	−0.411** (0.160)	1.963*** (0.037)	0.653*** (0.169)
Observations	8,599	8,599	8,599	8,599	8,599	8,599

***, **, and * indicate significance at the 1, 5, and 10% levels, respectively; robust standard errors are in parentheses.

**Table 4:** The impact of CHE on individuals’ social identity perceptions.

Variables	(1)		(2)		(3)	
	Subjective well-being		Life satisfaction		Social trust	
Catastrophic health expenditure	−0.183*** (0.048)	−0.205*** (0.050)	−0.441*** (0.046)	−0.340*** (0.048)	−0.154*** (0.045)	−0.165*** (0.046)
Gender		0.171*** (0.044)		−0.020 (0.043)		0.074* (0.041)
Educational level		−0.015 (0.012)		0.064*** (0.012)		0.019* (0.011)
Age		0.014*** (0.002)		0.007*** (0.002)		0.006*** (0.002)
Marital status		−0.143*** (0.034)		−0.090*** (0.029)		−0.082*** (0.029)
Ethnicity		−0.461*** (0.083)		−0.293*** (0.081)		−0.312*** (0.081)
Household registration type		−0.068** (0.028)		−0.040 (0.027)		−0.060** (0.026)
Total income		0.000*** (0.000)		0.000*** (0.000)		0.000 (0.000)
/cut1	−3.624*** (0.070)	−3.520*** (0.184)	−3.598*** (0.066)	−3.294*** (0.174)	−3.515*** (0.066)	−3.578*** (0.166)
/cut2	−1.994*** (0.036)	−1.885*** (0.170)	−2.843*** (0.048)	−2.535*** (0.167)	−2.580*** (0.045)	−2.642*** (0.160)
/cut3	0.588*** (0.027)	0.731*** (0.166)	−1.327*** (0.030)	−0.998*** (0.161)	−0.829*** (0.028)	−0.889*** (0.155)
/cut4			−0.102*** (0.026)	0.252 (0.160)	0.561*** (0.027)	0.506*** (0.155)
Observations	8,599	8,599	8,599	8,599	8,599	8,599

***, **, and * indicate significance at the 1, 5, and 10% levels, respectively; robust standard errors are in parentheses.

As Tables 3, 4 show, when the control variables are included, the coefficients for perceptions of public medical equity, perceptions of social security equity, perceptions of urban–rural equity, subjective well-being, life satisfaction, and social trust are −0.107, −0.132, −0.214, −0.205, −0.340, and −0.165, respectively. The estimated CHE on individuals’ perceptions of public medical equity is significantly negative at the 5% level, while the estimated coefficients on all indicators of perceptions of social security equity, perceptions of urban–rural equity, and social identity are all significantly negative at the 1% level. This indicates that individuals with CHE have significantly lower perceptions of social equity and social identity compared with those without CHE. In addition, a comparison of the coefficients reveals that CHE exerts a stronger impact on the three dimensions of individuals’ social identity. These findings verify Hypotheses 1 and 3, demonstrating that the occurrence of CHE exerts an adverse impact on social stability.

#### The impact of CHE intensity on social stability

Given that individuals with CHE exhibit lower perceptions of social equity and social identity, this study further explores the impact of CHE intensity on individuals’ perceptions of equity in public medical care, social security, and urban–rural benefits, as well as on their subjective well-being, life satisfaction, and social trust. The results are presented in Tables 5, 6.

**Table 5:** The impact of CHE intensity on individuals’ perceptions of social equity.

Variables	(1)		(2)		(3)	
	Public healthcare equity perception		Social security equity perception		Urban–rural equity perception	
Catastrophic health expenditure intensity	−0.468*** (0.141)	−0.323** (0.142)	−0.460*** (0.142)	−0.292** (0.144)	−0.792*** (0.140)	−0.559*** (0.140)
Gender		0.124*** (0.044)		−0.018 (0.042)		0.028 (0.042)
Educational level		−0.024** (0.012)		−0.035*** (0.012)		−0.011 (0.012)
Age		−0.015*** (0.002)		−0.019*** (0.002)		−0.017*** (0.002)
Marital status		−0.127*** (0.032)		−0.028 (0.030)		−0.010 (0.029)
Ethnicity		−0.905*** (0.087)		−0.965*** (0.084)		−0.809*** (0.092)
Household registration type		−0.057** (0.028)		−0.020 (0.026)		0.081*** (0.026)
Total income		−0.000 (0.000)		−0.000 (0.000)		0.000 (0.000)
/cut1	−2.938*** (0.049)	−4.735*** (0.175)	−2.231*** (0.037)	−4.290*** (0.168)	−1.773*** (0.033)	−3.159*** (0.171)
/cut2	−1.217*** (0.029)	−2.994*** (0.169)	−0.833*** (0.026)	−2.868*** (0.164)	−0.208*** (0.025)	−1.566*** (0.168)
/cut3	1.388*** (0.030)	−0.315* (0.164)	1.564*** (0.031)	−0.397** (0.159)	1.985*** (0.036)	0.672*** (0.168)
Observations	8,599	8,599	8,599	8,599	8,599	8,599

***, **, and * indicate significance at the 1, 5, and 10% levels, respectively; robust standard errors are in parentheses.

**Table 6:** The impact of CHE intensity on individuals’ social identity perceptions.

Variables	(1)		(2)		(3)	
	Happiness		Life Satisfaction		Social Trust	
Catastrophic health expenditure intensity	−0.554*** (0.150)	−0.630*** (0.152)	−1.261*** (0.138)	−0.980*** (0.142)	−0.574*** (0.132)	−0.608*** (0.135)
Gender		0.169*** (0.044)		−0.024 (0.043)		0.073* (0.041)
Educational level		−0.015 (0.012)		0.063*** (0.012)		0.018 (0.011)
Age		0.014*** (0.002)		0.007*** (0.002)		0.006*** (0.002)
Marital status		−0.141*** (0.034)		−0.088*** (0.029)		−0.081*** (0.029)
Ethnicity		−0.464*** (0.083)		−0.299*** (0.081)		−0.314*** (0.081)
Household registration type		−0.066** (0.028)		−0.036 (0.027)		−0.059** (0.026)
Total income		0.000*** (0.000)		0.000*** (0.000)		0.000 (0.000)
/cut1	−3.614*** (0.069)	−3.507*** (0.183)	−3.568*** (0.065)	−3.272*** (0.173)	−3.517*** (0.065)	−3.576*** (0.166)
/cut2	−1.984*** (0.035)	−1.872*** (0.170)	−2.812*** (0.047)	−2.512*** (0.166)	−2.581*** (0.044)	−2.640*** (0.160)
/cut3	0.599*** (0.026)	0.744*** (0.166)	−1.295*** (0.028)	−0.974*** (0.161)	−0.829*** (0.026)	−0.886*** (0.155)
/cut4			−0.070*** (0.025)	0.276* (0.159)	0.562*** (0.025)	0.511*** (0.154)
Observations	8,599	8,599	8,599	8,599	8,599	8,599

***, **, and * indicate significance at the 1, 5, and 10% levels, respectively; robust standard errors are in parentheses.

As shown in Tables 5, 6, after the inclusion of control variables, the coefficients of CHE intensity on individuals’ perceptions of public medical equity, social security equity, urban–rural rights equity, subjective well-being, life satisfaction, and social trust are −0.323, −0.292, −0.559, −0.630, −0.980, and −0.608, respectively. Among these coefficients, those for perceptions of public medical equity and social security equity are significant at the 5% level, while all the others are significant at the 1% level. This indicates that overall, an increase in CHE intensity leads to a significant decrease in individuals’ perceptions of social equity and social identity, thus verifying Hypotheses 2 and 4. It can be concluded that a rise in CHE intensity will further undermine social stability.

### Robustness tests

#### Heckman test

The results presented in the baseline regression may be driven by the inherent characteristics of the sample rather than by CHE and its intensity. To address the issues of sample self-selection bias and potential non-random missing data in the data processing process, and considering that CHE and CHE intensity involved in the baseline regression are a binary variable and a continuous variable, respectively, this study draws on existing research and employs the Heckman two-stage model to handle the problem of selection bias (52).

First, a probit model is used to estimate the sample selection probabilities of CHE occurrence and CHE intensity, respectively. The overall CHE incidence rate at the city/county/district level is closely associated with factors such as the economic and medical security levels of the region where an individual resides, and thus exerts an impact on an individual’s CHE status. However, the group-level CHE incidence rate does not directly affect an individual’s micro-level perceptions of social equity and social identity. Therefore, this variable is selected as an exogenous variable for regression to generate the Inverse Mills Ratio (IMR). The IMR is then incorporated into the baseline regression as an additional control variable for further analysis, with the results presented in Tables 7, 8.

**Table 7:** Heckman test: the impact of CHE on individuals’ perceptions of social equity and social identity.

Variables	(1)	(2)	(3)	(4)	(5)	(6)
	Public healthcare equity perception	Social security equity perception	Urban–rural equity perception	Happiness	Life Satisfaction	Social Trust
Catastrophic health expenditure	−0.124** (0.050)	−0.154*** (0.048)	−0.216*** (0.047)	−0.226*** (0.050)	−0.339*** (0.048)	−0.156*** (0.047)
IMR	−0.219* (0.117)	−0.290** (0.114)	−0.021 (0.113)	−0.272** (0.119)	0.007 (0.116)	0.111 (0.109)

Other control variables	Yes					
/cut1	−5.054*** (0.240)	−4.719*** (0.232)	−3.209*** (0.232)	−3.907*** (0.247)	−3.284*** (0.241)	−3.420*** (0.229)
/cut2	−3.312*** (0.236)	−3.296*** (0.228)	−1.616*** (0.229)	−2.272*** (0.238)	−2.525*** (0.236)	−2.484*** (0.224)
/cut3	−0.633*** (0.232)	−0.823*** (0.225)	0.624*** (0.229)	0.345 (0.235)	−0.988*** (0.232)	−0.731*** (0.221)
/cut4					0.262 (0.231)	0.664*** (0.220)
Observations	8,599	8,599	8,599	8,599	8,599	8,599

***, **, and * indicate significance at the 1, 5, and 10% levels, respectively; robust standard errors are in parentheses.

**Table 8:** Heckman test: the impact of CHE intensity on individuals’ perceptions of social equity and social identity.

Variables	(1)	(2)	(3)	(4)	(5)	(6)
	Public healthcare equity perception	Social security equity perception	Urban–rural equity perception	Happiness	Life Satisfaction	Social Trust
Catastrophic health expenditure intensity	−0.359** (0.143)	−0.340** (0.145)	−0.558*** (0.141)	−0.677*** (0.154)	−0.972*** (0.143)	−0.588*** (0.137)
IMR	−0.207* (0.116)	−0.263** (0.114)	0.009 (0.113)	−0.256** (0.119)	0.041 (0.115)	0.111 (0.108)

Other control variables	Yes					
/cut1	−5.028*** (0.238)	−4.664*** (0.231)	−3.147*** (0.230)	−3.870*** (0.246)	−3.213*** (0.239)	−3.420*** (0.227)
/cut2	−3.287*** (0.234)	−3.242*** (0.227)	−1.553*** (0.227)	−2.234*** (0.237)	−2.453*** (0.234)	−2.483*** (0.222)
/cut3	−0.608*** (0.230)	−0.770*** (0.224)	0.685*** (0.227)	0.383 (0.234)	−0.915*** (0.230)	−0.729*** (0.219)
/cut4					0.335 (0.229)	0.668*** (0.218)
Observations	8,599	8,599	8,599	8,599	8,599	8,599

***, **, and * indicate significance at the 1, 5, and 10% levels, respectively; robust standard errors are in parentheses.

From the results of the Heckman test, among the six selected indicators, the coefficients of the IMR are only significant for perceptions of public medical equity, social security equity, and subjective well-being, indicating the existence of a certain degree of sample selection bias. However, after incorporating the IMR into the regression, the coefficients of CHE and CHE intensity on individuals’ perceptions of public medical equity, social security equity, urban–rural rights equity, subjective well-being, life satisfaction, and social trust remain significantly negative at the 5% level. This suggests that after excluding sample selection errors, both the occurrence of CHE and an increase in CHE intensity lead to a decrease in individuals’ perceptions of social equity and social identity, exerting an adverse impact on social stability, which verifies the robustness of the baseline regression conclusions.

#### Changing the method of calculating the explanatory variable

In the baseline regression, the criterion for identifying CHE is defined as out-of-pocket medical expenses accounting for 10% of total household expenditure. Some scholars have empirically demonstrated that in China, the CHE threshold should be set at 44.13% of annual household out-of-pocket medical expenses relative to household income (53). Accordingly, other scholars adopt 40% of out-of-pocket medical expenses to household income as the CHE threshold. Based on this, a robustness test is conducted by changing the calculation method of the explanatory variable: households with out-of-pocket medical expenses accounting for 40% or more of per capita household income are identified as having incurred CHE and coded as 1, otherwise 0; for households with out-of-pocket medical expenses below 40% of household income, their CHE intensity is regarded as 0, while for other households, CHE intensity is equivalent to the proportion of their out-of-pocket medical expenses to household income. The regression results after adjusting the calculation method of the explanatory variable are presented in Tables 9, 10.

**Table 9:** Robustness test: the impact of CHE on perceptions of social equity and social identity.

Variables	(1)	(2)	(3)	(4)	(5)	(6)
	Public healthcare equity perception	Social security equity perception	Urban–rural equity perception	Happiness	Life satisfaction	Social trust
Catastrophic health expenditure	−0.376*** (0.085)	−0.380*** (0.085)	−0.370*** (0.082)	−0.546*** (0.100)	−0.845*** (0.083)	−0.385*** (0.082)

Control variables	Yes					
/cut1	−4.750*** (0.175)	−4.308*** (0.168)	−3.158*** (0.171)	−3.522*** (0.183)	−3.318*** (0.172)	−3.573*** (0.166)
/cut2	−3.007*** (0.169)	−2.883*** (0.164)	−1.565*** (0.168)	−1.886*** (0.169)	−2.558*** (0.165)	−2.637*** (0.159)
/cut3	−0.325** (0.164)	−0.410** (0.160)	0.674*** (0.168)	0.723*** (0.166)	−1.021*** (0.159)	−0.883*** (0.155)
/cut4					0.221 (0.158)	0.514*** (0.154)
Observations	8,599	8,599	8,599	8,599	8,599	8,599

***, **, and * indicate significance at the 1, 5, and 10% levels, respectively; robust standard errors are in parentheses.

**Table 10:** The impact of CHE intensity on perceptions of social equity and social identity.

Variables	(1)	(2)	(3)	(4)	(5)	(6)
	Public healthcare equity perception	Social security equity perception	Urban–rural equity perception	Happiness	Life satisfaction	Social trust
Catastrophic health expenditure intensity	−0.410*** (0.103)	−0.463*** (0.103)	−0.408*** (0.099)	−0.595*** (0.129)	−0.880*** (0.109)	−0.425*** (0.105)

Control variables	Yes					
/cut1	−4.742*** (0.174)	−4.304*** (0.168)	−3.152*** (0.171)	−3.502*** (0.183)	−3.271*** (0.173)	−3.563*** (0.165)
/cut2	−3.000*** (0.169)	−2.880*** (0.164)	−1.559*** (0.168)	−1.864*** (0.170)	−2.509*** (0.166)	−2.627*** (0.159)
/cut3	−0.319* (0.164)	−0.407** (0.159)	0.679*** (0.168)	0.754*** (0.166)	−0.966*** (0.160)	−0.873*** (0.155)
/cut4					0.286* (0.159)	0.523*** (0.154)
Observations	8,599	8,599	8,599	8,599	8,599	8,599

***, **, and * indicate significance at the 1, 5, and 10% levels, respectively; robust standard errors are in parentheses.

The results show that after changing the calculation method of the explanatory variable, in which households with annual out-of-pocket medical expenses accounting for 40% of total annual household income are defined as incurring CHE, the estimated results of the corresponding variables for CHE and CHE intensity remain consistent with those of the baseline regression. This indicates that the core conclusions are still robust after adjusting the threshold for identifying CHE. The occurrence of CHE and the increase in its intensity reduce residents’ perceptions of social equity and social identity, and undermine social stability, which verifies the stability of the baseline regression results.

### Heterogeneity analysis

In the baseline regression, it has been confirmed that an increase in CHE intensity reduces individuals’ perceptions of social equity and social identity, thereby exerting a negative impact on social stability. To further analyze the differences in social equity perceptions and social identity among individuals bearing different out-of-pocket expense burdens, and to explore at which level the out-of-pocket medical expense burden imposes the strongest shock on social stability, this study conducts grouped comparisons based on the CHE burden. First, the sample is divided into two groups according to whether CHE occurs: the group without CHE is assigned a value of 0, i.e., individuals with out-of-pocket medical expenses accounting for less than 0.1 of total household expenditure are coded as 0. Since this study measures CHE as the ratio of out-of-pocket medical expenses to total household expenditure, the maximum value of CHE cannot exceed 1. Therefore, the group with CHE is further divided into two equidistant subgroups: the low-burden group [0.1, 0.55] and the high-burden group [0.55, 1]. Samples belonging to a given group are coded as 1, and 0 otherwise. Finally, three groups are formed: no burden, low burden, and high burden. Regression analyses are then performed separately with indicators of social equity perceptions and social identity. The results are presented in Table 11.

**Table 11:** Regression results of the three-way classification of CHE burden.

Variables	(1)	(2)	(3)	(4)	(5)	(6)
	Public healthcare equity perception	Social security equity perception	Urban–rural equity perception	Happiness	Life Satisfaction	Social Trust
[0.1, 0.55]	−0.099* (0.051)	−0.121** (0.049)	−0.200*** (0.047)	−0.223*** (0.051)	−0.367*** (0.049)	−0.152*** (0.047)
[0.55, 1]	−0.169 (0.126)	−0.203 (0.129)	−0.361*** (0.128)	−0.398*** (0.142)	−0.665*** (0.128)	−0.328*** (0.125)

Control variables	Yes					
/cut1	−4.739*** (0.175)	−4.303*** (0.168)	−3.178*** (0.172)	−3.537*** (0.183)	−3.332*** (0.173)	−3.580*** (0.166)
/cut2	−2.998*** (0.169)	−2.880*** (0.164)	−1.584*** (0.168)	−1.903*** (0.169)	−2.574*** (0.166)	−2.644*** (0.160)
/cut3	−0.320* (0.164)	−0.408** (0.160)	0.656*** (0.169)	0.703*** (0.165)	−1.044*** (0.160)	−0.891*** (0.155)
/cut4					0.195 (0.159)	0.504*** (0.154)
Observations	8,599	8,599	8,599	8,599	8,599	8,599

***, **, and * indicate significance at the 1, 5, and 10% levels, respectively; robust standard errors are in parentheses.

The results show that compared with individuals without CHE, those with an out-of-pocket medical expense burden in the interval [0.1, 0.55] have significantly lower perceptions of social equity and social identity, with coefficients of −0.099, −0.121, −0.200, −0.223, −0.367, and −0.152, respectively. The coefficient for public medical equity is significantly negative at the 10% level, that for social security equity is significant at the 5% level, and all others are significantly negative at the 1% level. When the share of out-of-pocket medical expenses exceeds 0.55, the high-burden group still exhibits significantly lower urban–rural rights equity, subjective well-being, life satisfaction, and social trust, with larger coefficients than the low-burden group.

Based on the three-group classification, this study further explores whether perceptions of social equity and social identity continue to decline as the share of out-of-pocket medical expenses rises. A four-group classification is adopted for regression. Specifically, samples without CHE are assigned a value of 0. The group with CHE is further divided into three equidistant subgroups according to the ratio of out-of-pocket expenses to total expenditure: [0.1, 0.4], [0.4, 0.7], and [0.7, 1], defined as the low-burden, medium-burden, and high-burden groups, respectively. Samples belonging to a given group are coded as 1, and 0 otherwise. The regression results are shown in Table 12.

**Table 12:** Regression results of the four-way CHE burden.

Variables	(1)	(2)	(3)	(4)	(5)	(6)
	Public healthcare equity perception	Social security equity perception	Urban–rural equity perception	Happiness	Life satisfaction	Social trust
[0.1, 0.4]	−0.088 (0.054)	−0.114** (0.052)	−0.195*** (0.049)	−0.169*** (0.053)	−0.283*** (0.051)	−0.113** (0.049)
[0.4, 0.7]	−0.237** (0.100)	−0.291*** (0.099)	−0.297*** (0.099)	−0.423*** (0.110)	−0.669*** (0.106)	−0.379*** (0.098)
[0.7, 1]	−0.032 (0.205)	0.124 (0.223)	−0.305 (0.203)	−0.138 (0.220)	−0.252 (0.193)	−0.385** (0.188)

Control variables	Yes					
/cut1	−4.743*** (0.175)	−4.308*** (0.168)	−3.178*** (0.172)	−3.520*** (0.184)	−3.298*** (0.174)	−3.577*** (0.166)
/cut2	−3.002*** (0.169)	−2.885*** (0.164)	−1.584*** (0.168)	−1.885*** (0.170)	−2.538*** (0.167)	−2.641*** (0.160)
/cut3	−0.322* (0.164)	−0.413*** (0.160)	0.656*** (0.169)	0.732*** (0.166)	−0.999*** (0.161)	−0.887*** (0.155)
/cut4					0.253 (0.160)	0.510*** (0.154)
Observations	8,599	8,599	8,599	8,599	8,599	8,599

***, **, and * indicate significance at the 1, 5, and 10% levels, respectively; robust standard errors are in parentheses.

According to the results in Table 12, overall, individuals with an out-of-pocket medical expense burden in the [0.4, 0.7] interval have significantly lower perceptions of social equity and social identity than those in the no-burden, low-burden, and high-burden groups. When the proportion of out-of-pocket medical expenses in total household expenditure exceeds 0.4, individuals’ perceptions of social equity and social identity decrease significantly. When the burden falls within [0.7, 1], there is no significant change in perceptions of social equity and social identity. That is, after the proportion of out-of-pocket medical expenses rises to a certain level, individuals’ perceptions of social equity and social identity no longer change significantly.

Within a certain range of out-of-pocket burden, perceptions of social equity and social identity decline as the individual burden increases. Combining the two classification methods, it can be inferred that when the proportion of out-of-pocket medical expenses to total household expenditure is in the interval [0.4, 0.55], the impact on all indicators of social stability is more significant.

### Cause analysis

According to the regression results, CHE leads to a decline in individuals’ social fairness perceptions and social identity perceptions, which are key factors influencing social stability. Consequently, the occurrence of CHE exerts an adverse impact on social stability. It is therefore imperative to further deepen the research and clarify the factors contributing to CHE, so as to avoid its occurrence at the source.

CHE occurs when individuals’ out-of-pocket medical expenses exceed a certain threshold due to the sustained utilization of medical services. If individuals have access to a high level of medical security benefits, their risk of incurring CHE will be reduced. Thus, it can be inferred that CHE is associated with medical service prices and the type of medical insurance in which individuals are enrolled. Meanwhile, CHE is closely linked to the health resource endowments of individuals and their families. Individuals with lower income levels and poorer access to medical services tend to face a higher risk of CHE (4). On this basis, the type of medical insurance enrollment, income level, access to medical services, and medical service prices are selected for separate analysis. The regression results are presented in Table 13.

**Table 13:** The impact of insurance type, income level, service accessibility and service price on CHE.

Variables	Catastrophic health expenditure			
	(1)	(2)	(3)	(4)
Urban employees’ basic medical insurance	−0.193** (0.085)			
Urban residents’ basic medical insurance	0.019 (0.086)			
Public medical services	0.236 (0.183)			
New rural cooperative medical scheme	0.220*** (0.071)			
Critical illness insurance	−0.161 (0.142)			
Others	0.197 (0.188)			

Control variables	Yes			
Income levels		−0.220*** (0.019)		
Healthcare accessibility			−0.263*** (0.032)	
Healthcare service prices				−0.238*** (0.026)
/cut1		1.007*** (0.190)	0.403* (0.231)	0.570*** (0.218)
Constant	−1.722*** (0.205)			
Observations	8,599	8,599	6,989	6,989

***, **, and * indicate significance at the 1, 5, and 10% levels, respectively; robust standard errors are in parentheses; Since some individuals in the sample did not use medical services in the past year, the sample size in Column (3) and Column (4) is 6,989.

The results show that the coefficients of Urban Employee Basic Medical Insurance and New Rural Cooperative Medical Scheme on CHE are both significant at the 5% level, while no significant correlation is found between other medical insurance types and CHE. Specifically, compared with uninsured individuals, those enrolled in Urban Employee Basic Medical Insurance can significantly reduce their risk of CHE, whereas individuals with the New Rural Cooperative Medical Scheme face a higher risk of CHE. Enrollment in Urban Resident Basic Medical Insurance, public medical services, critical illness insurance, and other medical insurance types has no significant effect on whether individuals incur CHE. The coefficients of individual income level, access to medical services, and medical service prices on CHE are all significant at the 1% level. This indicates that the lower the individual income level, the poorer the access to medical services, and the higher the medical service prices, the greater the risk of CHE. Income level, access to medical services, and medical service prices are thus important factors affecting the occurrence of CHE.

Based on the above analysis, this study further examines the impacts of insurance type, individual income level, access to medical services, and medical service prices on the intensity of CHE. Since only individuals who incur CHE are defined as having CHE intensity in this study, a large number of zero observations exist in the dependent variable. Left censoring of the dependent variable affects the probability density distribution; therefore, the Tobit model is used for regression. The average marginal effects are presented in Table 14.

**Table 14:** The impact of insurance type, income level, service accessibility and service price on CHE intensity.

Variables	Catastrophic health expenditure intensity			
	(1)	(2)	(3)	(4)
Urban employees’ basic medical insurance	−0.081*** (0.018)			
Urban residents’ basic medical insurance	−0.012 (0.019)			
Public medical services	0.014 (0.038)			
New rural cooperative medical scheme	0.046*** (0.016)			
Critical illness insurance	−0.037 (0.032)			
Others	0.027 (0.040)			

Control variables	Yes			
Income levels		−0.051*** (0.004)		
Healthcare accessibility			−0.056*** (0.006)	
Healthcare service prices				−0.051*** (0.005)
Observations	8,599	8,599	6,989	6,989

***, **, and * indicate significance at the 1, 5, and 10% levels, respectively; robust standard errors are in parentheses; Since some individuals in the sample did not use medical services in the past year, the sample size in Column (3) and Column (4) is 6,989.

According to Table 14, the coefficient of Urban Employee Basic Medical Insurance on CHE intensity is −0.081, while that of the New Rural Cooperative Medical Scheme (NCMS) is 0.046, which is significantly positive at the 1% level. This indicates that, compared with other medical insurance schemes, Urban Employee Basic Medical Insurance can significantly reduce CHE intensity, whereas NCMS tends to increase it.

This is because, relative to other medical insurance schemes, Urban Employee Basic Medical Insurance provides a relatively high level of benefits, which effectively reduces individuals’ out-of-pocket medical burden and further lowers their risk of catastrophic health expenditure. In contrast, NCMS offers lower benefit levels than employee medical insurance. In addition, individuals covered by NCMS usually have lower income and poorer health endowments. Constrained by differential reimbursement policies and limited affordability of medical expenses, they tend to seek medical care in clinics or primary healthcare institutions. On the one hand, such institutions are characterized by relatively low service quality and limited drug supply. After receiving treatment at these facilities, patients often have to purchase medicines at their own expense, seek referrals to higher-level hospitals, or receive cross-regional medical care, leading to higher total medical costs and fragmented healthcare-seeking behaviors, which prevent them from fully utilizing the NCMS reimbursement policy. On the other hand, some clinics are not designated medical institutions, making them ineligible for medical insurance reimbursement and thus increasing patients’ out-of-pocket expenses.

Moreover, the uninsured population is generally in better health, with less healthcare utilization and lower medical expenditures, which contributes to the counterintuitive result that NCMS enrollees face a higher risk of CHE. Overall, apart from Urban Employee Basic Medical Insurance, other types of medical insurance do not exert significant effects on reducing individuals’ medical burden or mitigating CHE risk, revealing the existence of internal imbalance and insufficiency in the medical security system.

The coefficients of individual income level, access to medical services, and medical service prices on CHE intensity are all significant at the 1% level. This indicates that higher income levels are associated with lower CHE intensity; better access to medical services reduces CHE intensity; and higher medical service prices increase CHE intensity. Individuals and households with higher income levels usually possess greater economic affordability and better health resource endowments. Given the same medical service prices and healthcare demands, the proportion of out-of-pocket medical expenses relative to household payment capacity is relatively lower. Therefore, higher income levels correspond to lower CHE intensity. In addition, higher accessibility to quality medical services helps reduce unnecessary medical expenditures such as cross-regional medical treatment and repeated visits. Medical service prices directly affect individuals’ medical expense burden. Accordingly, income level, access to medical services, and medical service prices exert significant impacts on the intensity of individuals’ catastrophic health expenditure.

## Discussion

This study adopted an ordered logit model to analyze the impact of CHE on individuals’ perceptions of social equity and social identity. The results show that compared with individuals without CHE, those with CHE exhibit lower levels of social equity perception and social identity, indicating that CHE significantly reduces individuals’ perceptions of social equity and social identity and ultimately exerts an adverse effect on social stability.

Kockaya et al. (54) found that insufficient government investment in medical and health care would expose individuals to the risk of incurring CHE. Dizon revealed that in the face of CHE risk, security inequality would further undermine individuals’ subjective well-being; it is imperative to balance the allocation of medical insurance funds across different insurance schemes (55). China’s medical security system is plagued by the problem of imbalance in benefit protection. Except for the Urban Employee Basic Medical Insurance, all other types of medical insurance and public medical services suffer from inadequate protection. In particular, affected by the low security level and the particularity of the insured group, the NCMS even presents a phenomenon where the incidence of CHE is higher among its enrollees than the uninsured population. Li et al. (56) also confirmed that the NCMS fails to prevent the occurrence of CHE.

Against the backdrop of protection inequality, income disparities among individuals arising from the primary distribution of the market exacerbate the inequality in CHE risks across populations. As demonstrated in Pal’s research, an increase in income leads to a decrease in the incidence of CHE (57). Xu et al. (58) indicated that households with multidimensional borderline poverty face a far higher risk of CHE than those without such poverty. Individuals with low incomes have weak economic affordability, poor resource endowments, and access to only low-tier medical insurance benefits. Coupled with the combined impacts of high medical service prices and poor access to medical services, disadvantaged groups are more prone to incurring CHE, which reduces their perceptions of social equity and social identity and ultimately undermines social stability.

## Limitations of the study

This study has certain limitations. First, as the CSS database is a repeated cross-sectional database that only conducts follow-up surveys on some individuals in selected years, this study is also presented in the form of a cross-sectional analysis. It thus fails to better measure the dynamic changes in the impact of shifts in individuals’ CHE status and variations in CHE intensity on social stability. Second, while this study initially explores the effects of different levels of out-of-pocket medical expense burden on individuals’ perceptions of social equity and social identity, and roughly estimates the out-of-pocket expense burden interval that exerts the strongest impact on social stability, it does not conduct more in-depth research to obtain a more precise range of this interval. Finally, this study adopts individual-level data for measurement. In future research, attempts may be made to measure CHE using the overall incidence of catastrophic health expenditure at the provincial, municipal, and county levels, and conduct comparative studies across different regions.

## Conclusion

Based on the empirical results and causal analyses of this study, CHE reduces individuals’ social fairness perceptions and social identity perceptions, and ultimately undermines social stability. Accordingly, it is necessary to optimize the allocation of medical resources, adjust the structure of fiscal investment, reverse the heavy concentration of high-quality medical resources in developed urban areas, and promote the downward deployment of quality medical resources. Efforts should be made to empower primary healthcare systems through talent introduction and equipment renewal, thereby improving the equity and accessibility of high-quality medical services for individuals.

Meanwhile, the gaps in benefit levels across different programs and regions within the medical security system should be narrowed. The medical insurance benefit standards in regions with high CHE incidence should be raised to reduce individuals’ medical expense burden and lower the incidence of CHE. The medical insurance reimbursement policy should be improved by exploring the establishment of a unified out-of-pocket cap mechanism. Pilot reforms can start with out-of-pocket caps within the inpatient reimbursement catalogue of resident medical insurance and gradually shift toward caps on actual personal out-of-pocket expenses.

On this basis, cooperation with financial and tax authorities should be strengthened to build a resident income database, enabling medical insurance departments to better monitor residents’ income status. An exploration can be made to transform a uniform out-of-pocket cap into an income-related tiered cap system, with different ceilings set according to income groups and gradually refined to enhance targeting, with priority given to low-income and vulnerable groups. By controlling the level of personal out-of-pocket medical expenses and improving overall protection, CHE can be fundamentally prevented.

## Data Availability

The original contributions presented in the study are included in the article/supplementary material, further inquiries can be directed to the corresponding author/s.
